# Immunohistochemical identification of primary peritoneal serous cystadenocarcinoma mimicking advanced colorectal carcinoma: a case report

**DOI:** 10.1186/1752-1947-1-150

**Published:** 2007-11-26

**Authors:** Wesley B von Riedenauer, Sumbul A Janjua, David S Kwon, Ziying Zhang, Vic Velanovich

**Affiliations:** 1 Department of Surgery, Henry Ford Hospital, Detroit, Michigan, USA; 2 Department of Pathology, Henry Ford Hospital, Detroit, Michigan, USA; 3 The Aga Khan University Medical College, Karachi, Pakistan

## Abstract

Primary peritoneal cystadenocarcinoma is a rare tumor of similar histogenic origin as primary ovarian carcinoma. We present a case of primary peritoneal serous cystadenocarcinoma mimicking advanced colorectal cancer in a 68 yr-old African American female. Radiology, endoscopy and cytology yielded only inconclusive findings. Immunohistochemical analysis of percutaneously obtained ascitic fluid provided a correct diagnosis of primary peritoneal cystadenocarcinoma. The discovery of serous ascites at the time of laparotomy confirmed a diagnosis of primary peritoneal serous cystadenocarcinoma. Final surgical pathology reconfirmed the diagnosis of primary peritoneal cystadenocarcinoma. This case demonstrates the utility of immunohistochemistry for accurately diagnosing patients with inconclusive findings in the setting of peritoneal carcinomatosis and primary peritoneal cystadenocarcinoma.

## Introduction

Primary peritoneal cystadenocarcinoma is a rare tumor [1]. Originally described by Swerdlow in 1959, the true incidence of primary peritoneal cystadenocarcinoma remains unknown although an estimated relative frequency to ovarian cancer is 1:10[2]. Better recognition of this entity in recent years has contributed to an increasing diagnostic frequency approaching 18% of laparotomies performed for ovarian carcinoma [2]. Synonyms for primary peritoneal cystadenocarcinoma include primary peritoneal papillary carcinoma, extraovarian peritoneal papillary carcinoma, peritoneal mesothelioma, surface papillary carcinoma, primary peritoneal carcinoma, and multiple focal extraovarian carcinoma [1]. Primary ovarian carcinoma may present as a solitary mass, but is the most common cause of carcinomatosis in women. Primary peritoneal cystadenocarcinoma uniformly presents as disseminated intraperitoneal carcinomatosis.

Both primary ovarian carcinoma and primary peritoneal cystadenocarcinoma can present with carcinomatosis. Clinical presentation results from local tumor effects involving multiple organs [3]. Common symptoms include abdominal distension/ascites, dyspnea, nausea, vomiting and constipation. The most common presenting complaints are ascites, abdominal mass and pleural effusion. Both primary ovarian carcinoma and primary peritoneal cystadenocarcinoma have serous and mucinous subtypes. The serous subtypes, primary serous ovarian carcinoma and primary peritoneal serous cystadenocarcinoma, predominate over the mucinous subtypes in both primary ovarian carcinoma and primary peritoneal cystadenocarcinoma. Ascites and abdominal distension appear more frequently with primary peritoneal cystadenocarcinoma than with primary ovarian carcinoma. Primary ovarian carcinoma alternately presents more often with a palpable pelvic mass. Primary peritoneal cystadenocarcinoma and primary ovarian carcinoma are immunohistochemically indistinguishable [1,4]. Both malignancies stain positive for CK7, ER, Mesothelin and CA125. Primary peritoneal cystadenocarcinoma is histologically distinguished from primary ovarian carcinoma by ovaries of normal size or enlarged secondary to a benign process, extraovarian involvement greater than ovarian involvement, and ovarian surface penetration of less than 5 mm depth [1,3,4].

Management of primary peritoneal cystadenocarcinoma has followed treatment of primary ovarian carcinoma with surgical debulking and adjuvant platinum-containing chemotherapy [4]. Survival in primary peritoneal serous cystadenocarcinoma parallels survival of stage III–IV primary serous ovarian carcinoma [1,4,5]. Median survivals for primary peritoneal serous cystadenocarcinoma and primary serous ovarian carcinoma are equivalent and range from 32–40 months [1,5].

Primary peritoneal cystadenocarcinoma and primary ovarian carcinoma both stain positive for estrogen receptor (ER), cytokeratin 7 (CK7), Wilm's tumor suppressor gene (WT1), and cancer antigen 125 (CA 125). Neither entity possesses cytokeratin 20 (CK 20), progesterone receptor (PR), Calretinin, carcinoembryonic antigen (CEA), gross cystic disease fluid protein (BRST-2), and thyroid transcription factor 1 (TTF1). Cytochemical overlap can occur between different celltypes but the constellation of positive and negative antigenicity produces a unique immunohistochemical "fingerprint" that identifies cellular origin.

## Case presentation

**Figure 1:**
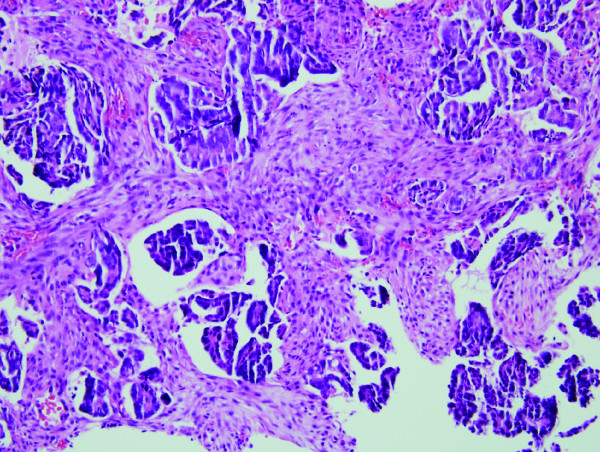
H & E section of sigmoid colonic mucosal biopsy demonstrating nests of infiltrating neoplastic glands with a papillary growth pattern.

The patient was a 68 yr-old African American female who presented to the emergency department of Henry Ford Hospital with complaints of shortness of breath and abdominal distension. Her past medical history was significant for asthma, Type II diabetes mellitus, hypertension, gastroesophageal reflux, adenomatous colonic polyps and breast cancer. Abdominal ascites and a right pleural effusion were present. Ultrasound imaging (US) of the abdomen and pelvis was normal except for the known ascites. Computed tomography (CT) of the abdomen and pelvis demonstrated moderate ascites, a right pleural effusion and omental thickening. US and CT imaging failed to demonstrate any masses. Magnetic resonance imaging of the abdomen and pelvis revealed pelvic peritoneal masses suspicious for metastatic implants and a 4.1 centimeters mass in the appendix. Colonoscopy was performed to the cecum. At 50 centimeters proximal to the anal verge, a 4 centimeters subserosal non-circumferential partially occluding mass was discovered. The mass was biopsied. The remainder of the exam was normal. Consideration was given to an endoscopically concealed appendiceal cystadenocarcinoma or primary appendiceal colonic adenocarcinoma. Pathology reported the endoscopic tissue biopsy as displaying a moderately well differentiated adenocarcinoma with a papillary growth pattern (Fig. 1). Attendant paracentesis was performed and the ascitic fluid obtained was positive for malignant cells consistent with metastatic adenocarcinoma. Immunohistochemical staining of the malignant ascitic cells was positive for CA 125, WT-1, CK7 and ER but negative for CK20, TTF-1, PR, Calretinin and BRST-2. These immunohistochemistry results were consistent with primary peritoneal cystadenocarcinoma or primary serous ovarian carcinoma. Ovarian origin was believed very unlikely without radiologic evidence of a pelvic mass. The gynecologic oncology service concurred that the probability of a primary ovarian origin was remote. Primary peritoneal cystadenocarcinoma became the operating diagnosis. Surgical consultation was requested.

**Figure 2:**
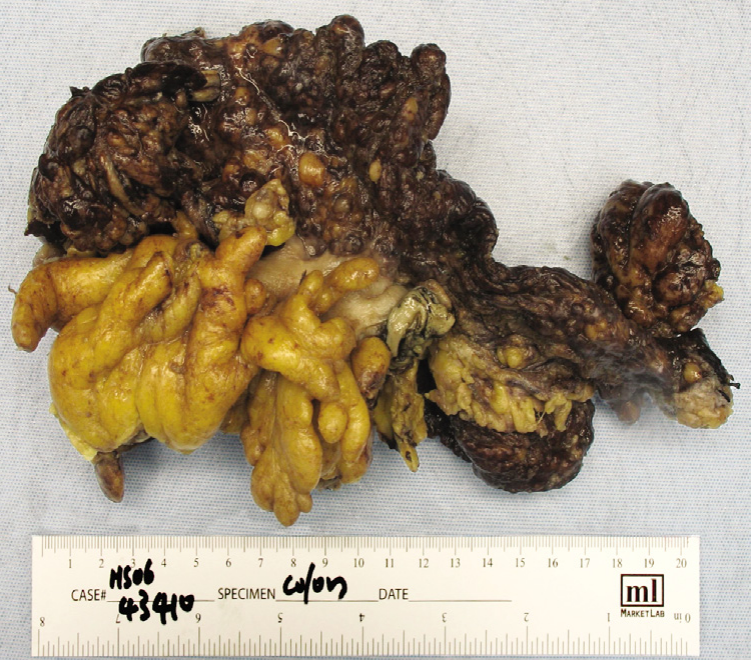
Gross picture of a segment of transverse colon with attached omentum. The Omentum is firm, nodular, and totally replaced by tumor.

**Figure 3:**
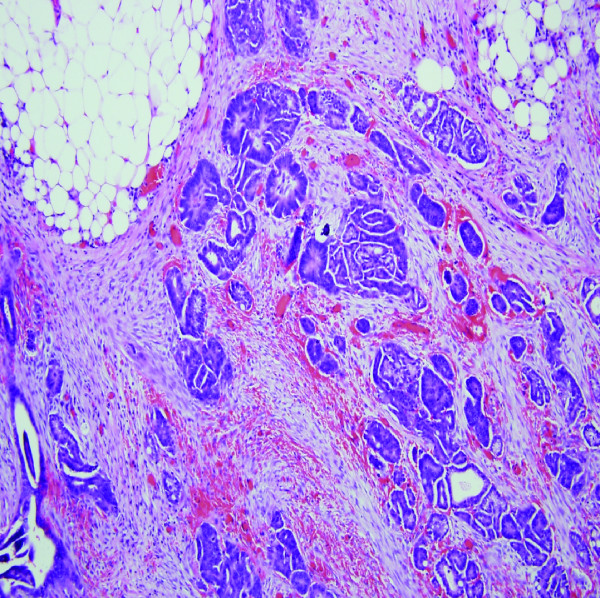
H & E section of omentum showing diffuse infiltration by tumor cells. The tumor cells demonstrate the papillary growth pattern characteristic of primary peritoneal adenocarcinoma.

Exploratory laparotomy was offered and performed. Penetration of the peritoneum immediately presented serous ascites. The omentum was grossly thickened and adherent to the mid transverse colon at 50 centimeters proximal to the anus. This section of transverse colon was segmentally excised (Fig. 2). Multiple areas of peritoneal studding significantly involved the small bowel gutter and ascending colon. Foreshortening of the cecal mesentery was noted in conjunction with a soft verrucous appendix. Effective debulking mandated a right hemicolectomy, which was performed. Intestinal continuity was re-established with a standard side-to-side ileocolostomy. Histologic evaluation of the submitted omentum and transverse colon specimen revealed a moderately differentiated adenocarcinoma with an infiltrative papillary growth pattern characteristic of primary peritoneal cystadenocarcinoma (Fig. 3). Histologic findings in the surgical and endoscopic biopsy specimens were analogous and confirmed primary peritoneal cystadenocarcinoma. These histologic findings also substantiated the immunohistochemical diagnosis of primary peritoneal cystadenocarcinoma. Postoperative ileus complicated the patient's recovery. She was discharged to home tolerating a regular diet on postoperative day 8. Systemic chemotherapy with carboplatin and Taxol was arranged in follow-up.

## Discussion

The patient presented with a diagnosis of metastatic adenocarcinoma of the colon. Her personal history of colonic polyps and her strong family history of colon cancer supported this diagnosis. Endoscopic and pathologic findings were inconsistent with advanced colorectal cancer. Radiologic imaging demonstrated peritoneal carcinomatosis but could not identify a unique origin. Accurately identifying the origin of the patient's peritoneal carcinomatosis predicated appropriate therapy as treatment differs among the varying malignancies causing peritoneal carcinomatosis. Disease progression and survival also differ among the varying malignancies causing peritoneal carcinomatosis and correct identification of malignant origin was required for accurate prognosis.

Breast and gastrointestinal metastasis may clinically, radiologically and biochemically mimic primary ovarian and primary peritoneal adenocarcinoma [6]. Peritoneal mesothelioma and pulmonary malignancies can also present clinically similar to primary ovarian carcinoma and primary peritoneal cystadenocarcinoma with abdominal pain and ascitic distension. Treatment of primary peritoneal mesothelioma and carcinomatosis from metastatic breast, lung and gastrointestinal cancers differ significantly from the treatment of primary ovarian and primary peritoneal adenocarcinoma. Surgical debulking with intraoperative hyperthermic chemotherapy provides some survival advantage with peritoneal mesothelioma [7]. Advanced peritoneal mesothelioma is treated with combination systemic chemotherapy [7]. Overall survival with peritoneal mesothelioma is less than twelve months [7]. Peritoneal carcinomatosis resulting from colorectal primaries has also been treated with intraoperative hyperthermic chemotherapy an surgical debulking [8]. Survival in patients with peritoneal carcinomatosis from colorectal primaries varies from 5.3 to 12.6 month [8]. Systemic chemotherapy for peritoneal carcinomatosis resulting from advanced colorectal cancer is 5-fluorouracil (5-FU) based with the addition of Oxaliplatin, Irinotecan, Bevacizumab, and Cetuximab based on the patient's performance status and whether prior chemotherapy was provided [9]. Median survival for peritoneal carcinomatosis secondary to disseminated pulmonary malignancies is 2 months [10]. Surgical intervention with breast cancer carcinomatosis shows a variable survival advantage [11]. Chemotherapy and hormonal manipulation prolongs survival with breast cancer carcinomatosis [11]. Cytoreductive surgery combined with systemic cisplatin-based multiagent chemotherapy extends survival in both primary ovarian and primary peritoneal cystadenocarcinoma [1]. Survival with carcinomatosis secondary to primary ovarian carcinoma and primary peritoneal cystadenocarcinoma can extend beyond thirty months [1].

Metastatic lung cancer and peritoneal mesothelioma were excluded based on the absence of pulmonary disease on radiologic imaging and the absence of characteristic histopathologic features on biopsy, respectively. The patient reported a personal history of breast cancer, *ductal carcinoma in situ*, treated with lumpectomy and radiation therapy five years prior. Surgical margins were negative. Tamoxifen was prescribed following completion of her radiation therapy, but was discontinued after one month. This history of breast cancer raised our suspicion of a breast origin. Metastatic breast cancer was considered unlikely given the rarity of peritoneal carcinomatosis from this disease in the absence of any other evidence of metastasis. Primary ovarian carcinoma was considered given the patient's personal history of breast cancer. Genetic testing was not performed. Consultation with the gynecologic oncology service disavowed a primary ovarian etiology. The possibility of primary peritoneal adenocarcinoma became prescient.

Immunohistochemical analysis lead to the correct identification of primary peritoneal cystadenocarcinoma while excluding alternate diagnoses. Tumor markers ordered included: CK7, CK20, TTF1, ER, PR, Calretinin, WT1, CA 125, BRST-2, and CEA. Colon adenocarcinoma stains positive for CK20 and typically negative for CK7, CA125, TTF1 and ER. Breast cancer cells typically stain positive for CK7, ER and are negative for CA125, CK20, and TTF1. Ovarian carcinoma binds antigens for CK7, CA125, ER, and is negative for CDX2, CK20, and TTF1. The patient's cells were immunohistochemically negative for CK20, TTF1, Calretinin, PR and BRST-2. Her immunohistochemistry was positive for CA125, WT1, CK7, and ER. Primary peritoneal cystadenocarcinoma bears antigens CK7, ER, WT1, and CA125. The constellation of symptoms, endoscopic morphology, imaging results, histo-pathologic and immunohistochemical findings portrayed primary peritoneal cystadenocarcinoma. Immunohistochemistry allowed the patient to be diagnosed without surgery. Without immunohistochemical technology, patients with diagnostically uncertain peritoneal carcinomatosis formerly required open or laparoscopic tissue biopsy with further operative interventions pending histologic analysis. Immunohistochemistry facilitated diagnosis without surgery.

Preoperatively discerning primary peritoneal serous cystadenocarcinoma from primary peritoneal mucinous adenocarcinoma was not possible as these two entities are indistinguishable immunohistochemically and histologically. Primary peritoneal serous cystadenocarcinoma was confirmed by the intraoperative finding of clear ascites and the patient was afforded significant tumor debulking. Histo-pathology returned a diagnosis of moderately differentiated invasive papillary adenocarcinoma, which was consistent with primary peritoneal cystadenocarcinoma. Our institution does not offer surgical debulking for peritoneal carcinomatosis secondary to advanced colorectal cancer. Persistent misdiagnosis of advanced colorectal cancer would have then resulted in suboptimal treatment of this patient by forgoing surgical debulking and assigning inappropriate chemotherapy.

## Conclusion

Cognizance of primary peritoneal cystadenocarcinoma and the utility of immunohistochemistry permitted the accurate diagnosis of a patient previously misidentified as having advanced colorectal adenocarcinoma. Correctly diagnosing primary peritoneal cystadenocarcinoma dramatically affected this patient's prognosis as opposed to metastatic colorectal adenocarcinoma with carcinomatosis. The incidence of primary peritoneal cystadenocarcinoma has increased dramatically over the past 10 years [2]. Perhaps the incident rise in primary peritoneal cystadenocarcinoma has resulted from a rising awareness of this disease and an increased ability to pathologically distinguish this disease. With the known ability of gastrointestinal malignancies to mimic gynecologic cancer, and with 1.1% of cancers referred to gynecologic oncologists reported to be nongynecologic [12], the ability to distinguish advanced colorectal cancer from primary peritoneal cystadenocarcinoma and primary ovarian carcinoma has become more important. Immunohistochemical technology demonstrated marked utility for distinguishing primary peritoneal cystadenocarcinoma from other mimicking diseases in the setting of unknown primary peritoneal carcinomatosis. This technology was successful using only paracentesis and thus obviated the risks and morbidity of surgical biopsy. Immunohistochemistry facilitated preoperative diagnostic certainty and thereby improved surgical planning. Improved patient outcomes may result from further use of immunohistochemical analysis to diagnose unknown primary peritoneal carcinomatosis.

## Competing interests

The author(s) declare that they have no competing interests.

## Authors' contributions

WV (Chief Resident/HOV) directly assembled patient information, directed further in depth analysis of the known literature, performed the operative interventions and managed the recovery of the patient, reviewed and directed several drafts of the manuscript, and was responsible for the final construction of the manuscript for submission to the senior staff/primary investigator.

SJ (MSIV) acquired patient information, researched the known literature, participated in the patient's surgery and recovery, and constructed the manuscript under guidance. In this capacity she directly contributed to the conceptualization, design and production of this manuscript. Her final manuscript was then submitted to senior members for final approval.

DK (HOIII) acquired patient information, instructed the junior investigator (SJ) in conceptualization and construction of the manuscript.

ZZ (Pathologist) processed, evaluated and diagnosed the submitted surgical specimens. She also constructed the pathology narrative for the final manuscript.

VV (Senior Staff) served as the senior staff reviewer/primary investigator on this case report. He initially conceived the possibility of alternative diagnoses, arranged patient testing, scheduled and immediately directed the surgical interventions, supervised the patient's recovery and follow-up. He was responsible for final approval of the submitted manuscript following directed revision.

## Consent

Written informed consent was obtained from the patient for publication of the study.
